# Assessment of the impact of social media addiction on psychosocial behaviour like depression, stress, and anxiety in working professionals

**DOI:** 10.1186/s40359-024-01850-2

**Published:** 2024-06-15

**Authors:** Vaishnavi Jahagirdar, Lenisha Ashlyn Sequeira, Nabeel Kinattingal, Tamsheel Fatima Roohi, Sultan Alshehri, Faiyaz Shakeel, Seema Mehdi

**Affiliations:** 1 Department of Pharmacology, JSS College of Pharmacy, JSS Academy of Higher Education & Research, Mysore, Karnataka 570015 India; 2 Department of Pharmaceutics, College of Pharmacy, King Saud University, Riyadh, 11451 Saudi Arabia

**Keywords:** Anxiety, Depression, Mental health, Psychological behaviours, Social media addiction, Stress

## Abstract

**Objective:**

Social media (SM), with its addictive nature and the accompanying psychosocial challenges such as stress, anxiety, and depression, is the primary factor exacerbating mental health problems and adversely impacting individuals’ wellbeing. Our study’s goal was to determine how SM affects employees’ psychosocial behaviours and assess the various factors that contributed to the employee’s excessive use of SM.

**Methods:**

A cross-sectional correlational analysis was conducted. Using a relevant questionnaire on employees, the study was assessed to establish the relationship or association between SM addiction and psychosocial disorders like depression, anxiety, and stress. 200 people with a minimum age of 24 were enrolled in the study. The questionnaire contained the social networking addiction scale (SNAS) and the depression, anxiety, and stress-21 (DASS-21) scales; the data were statistically assessed.

**Results:**

The association between SM addiction and psychosocial behaviours has been examined using statistical tools including descriptive statistics and the Chi-square analysis. SM addiction has a strong, statistically significant correlation with depression (*p* = 0.001), stress (*p* = 0.001), and anxiety (*p* = 0.001).

**Conclusion:**

This study discovered a connection between SM use and depression, stress, and anxiety among working employees, raising questions regarding worries about overuse and addiction to SM. Various factors influencing excessive usage included revealed that employees also majorly over used SM for entertainment, boredom avoidance, constant knowledge sharing, and relationship-building.

**Supplementary Information:**

The online version contains supplementary material available at 10.1186/s40359-024-01850-2.

## Introduction

A web-based tool called social media (SM) facilitates the development of social networks and interpersonal relationships between people who have similar experiences, passions, pursuits, and connections. The increase in the availability of gadgets like laptops, smartphones, and tablets is driving the use of SM networking sites. There is much potential for people to use these digital gadgets without regard to time or space constraints and virtually communicate [1]. Over 59% of the global population, totalling 4.76 billion people, engages in SM, with 137 million new users in the past year. The average daily time spent on these platforms is 2 h and 31 min as of January 2023. Over the last decade, browsing and swiping on social networking sites have become increasingly common. While SM use is largely harmless, a tiny proportion of Americans—between 5 and 10%—show addicted behaviours and use it obsessively [2] social media addiction (SMA) is characterized by excessive concern about SM, an insatiable urge to access or use SM, and a commitment of more time and energy to SM to the point that it interferes with other significant elements of life [3]. 36.6% of 16-24-year-olds spend their leisure time on SM, and 47.5% use it to communicate with friends and family. While 34.8% of users click on to read news stories, 31% of users log on to search for articles or videos.

Users use multiple social networks. Around the world, 7.4 platforms are utilized on a monthly average. The average number of SM platforms used per person is 8.7 in India, compared to 3.7 in Japan. Each platform is utilized for various purposes by users, Facebook (71.1%) for messaging, TikTok (77.4%) and Reddit (37.8%) for fun, and Snapchat (40.3%) and Instagram (69.9%) for sharing pictures and videos [4]. The average time a person spends on these channels daily is Facebook (33 min), LinkedIn (<1 min), Instagram (29 min), Whatsapp (28 min), Snapchat (31 min) and Twitter (31 min). Previous studies found that the main predictor of mental health problems was not age but rather gender, with women being far more likely than men to develop mental health issues [5].

**Fig. 1:**
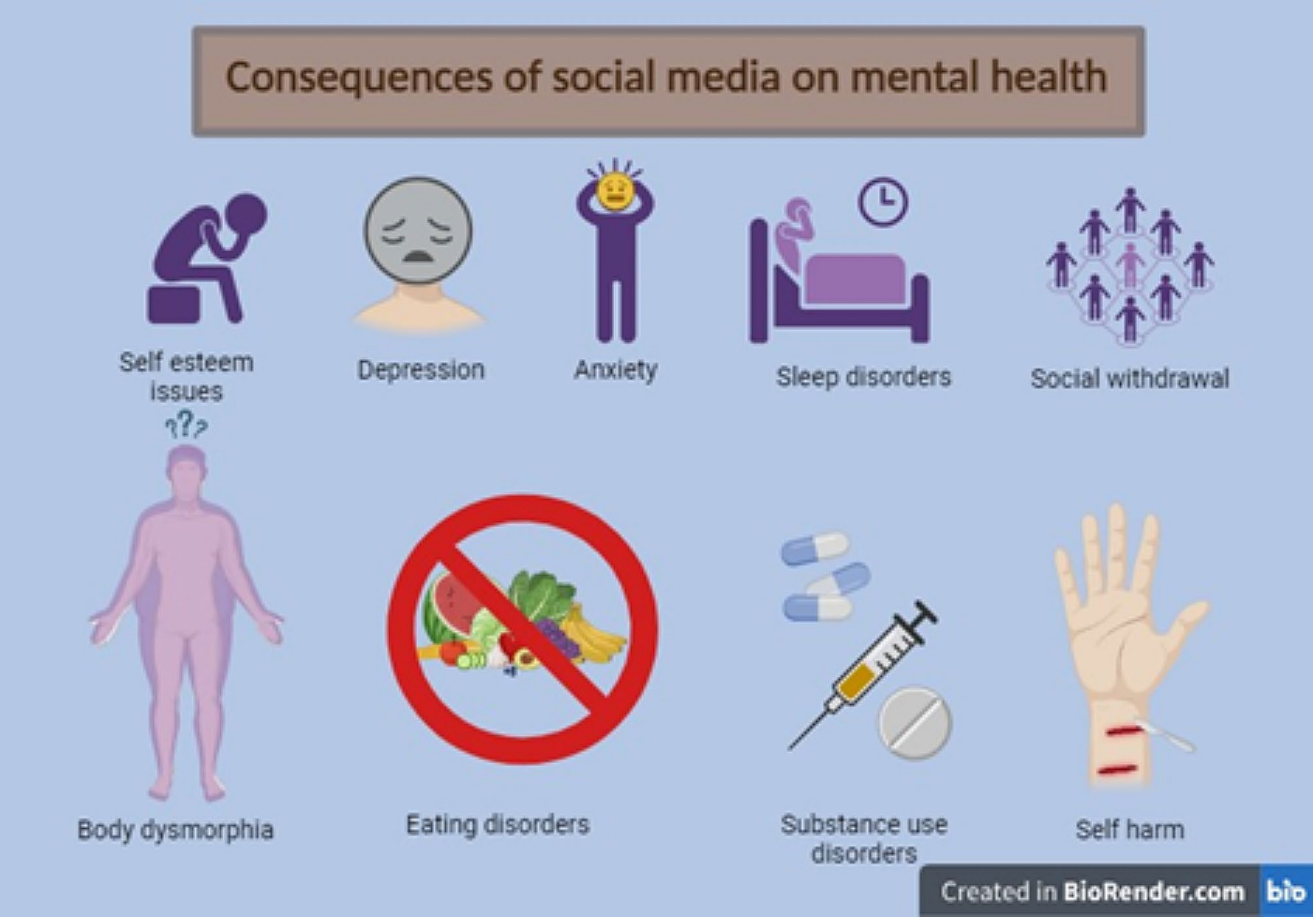
The impact of addiction to social media on employees’ physical and emotional welfare (Created with BioRender.com)

Excessive SM use is linked to participants’ fear of missing out (FOMO), the desire for up-to-date information, and the cycle of notifications [6]. The time lost as a result of this over-engagement is sometimes overlooked [7]. SMA among workers adversely affects productivity by causing them to miss deadlines, compromise the quality of their work, and become easily distracted from their tasks [8, 9]. Additionally damaging to their career and personal relationships, SMA makes people feel insecure and develop an inferiority complex [10, 11]. The impact of SMA on employees’ physical and emotional welfare are depicted in Fig. 1 [12]. Excessive SM use is increasingly recognized as a behavioural addiction, sharing similarities with other addictive disorders [13]. Due to the similarities such as withdrawal, conflict, relapse, tolerance, and mood alteration, excessive SM use has lately been considered a behavioural addiction [14, 15].

SM triggers dopamine, the ultimate feel-good chemical, in order to take advantage of our brain’s reward system. Our brains release the neurotransmitter dopamine whenever we have a happy experience. It’s how our brain remembers that engaging in certain activities will make us feel good, which encourages us to keep doing them. When we exercise, consume delicious cuisine, or get a notification that someone liked our photo, dopamine is released [16]. Our dopamine levels spike in response to a phone notification, which encourages us to check and use our phones longer than we otherwise would. The most extreme form of this drive is known by scientists as “phantom text syndrome”, in which a user perceives or hears a ringtone or alert even when none is there. The brain creates a false excuse—a phantom SMS—because it wants you to check your phone. This phenomenon bears resemblance to both addiction and wants [17]. It has been shown that there is a stronger correlation between depression and passively browsing SM posts as opposed to actively viewing them [18, 19].

Extended use of social networking sites such as Facebook may be associated with negative symptoms of depression, anxiety, and stress [20, 21]. Face-to-face interaction with others is necessary to release the hormones that lower stress and improve your mood, physical health, and outlook on life [22]. Ironically, for a platform designed to foster community, excessive use of SM can exacerbate mental health conditions like anxiety and depression and leave you feeling alone and isolated [23, 24].

This study aims to assess the impact of SMA on psychosocial behaviours including depression, stress, and anxiety in employees [97 teaching staffs (Lecturers, Assistant Professors, Associate Professors, and Professors and 108 non-teaching staffs), and identify the key factors influencing SMA characteristics.

## Methods

### Study design

The study conducted is a cross-sectional, correlational type of research. The study was conducted to assess the relationship or association of SMA with psychosocial problems like depression, anxiety, and stress using a suitable questionnaire, on employees/working professionals (97 teaching staffs and 108 non-teaching staffs) [25].

### Study site

The study was conducted at JSS Academy of Higher Education and Research (JSS AHER) in Mysuru, Karnataka, India.

### Participants

The study included *N* = 200 participants of both genders. Among them, there were 200 employees/working professionals (97 teaching staffs and 108 non-teaching staffs) of > 24 years. The sample size of 200 was calculated using the Slovin or Yamane formula, where the standard of deviation, *P* = 0.5; margin of error, e = 0.04; confidence interval is 95%; population size, *N* = 2500 was considered [26]. The enrollment was not based on the history of depression, stress, and anxiety. However, the depression, stress, and anxiety were evaluated based on SMA. Inclusion criteria included employees/working professionals of either gender (age > 24), and participants willing to give their consent and participate in the study. Exclusion criteria included employees suffering from chronic diseases like heart disease, asthma, and cancer. This study was carried out in accordance with the Declaration of Helsinki. All study procedures were approved by the Institutional Ethics Committee (IEC Registration ECR/387/Inst/KA/2023/RR-19) at JSS Medical College, Mysuru, and obtained informed consent from all employees which was included in the Google Forms depicted in supplementary materials.

### Procedures

**Fig. 2:**
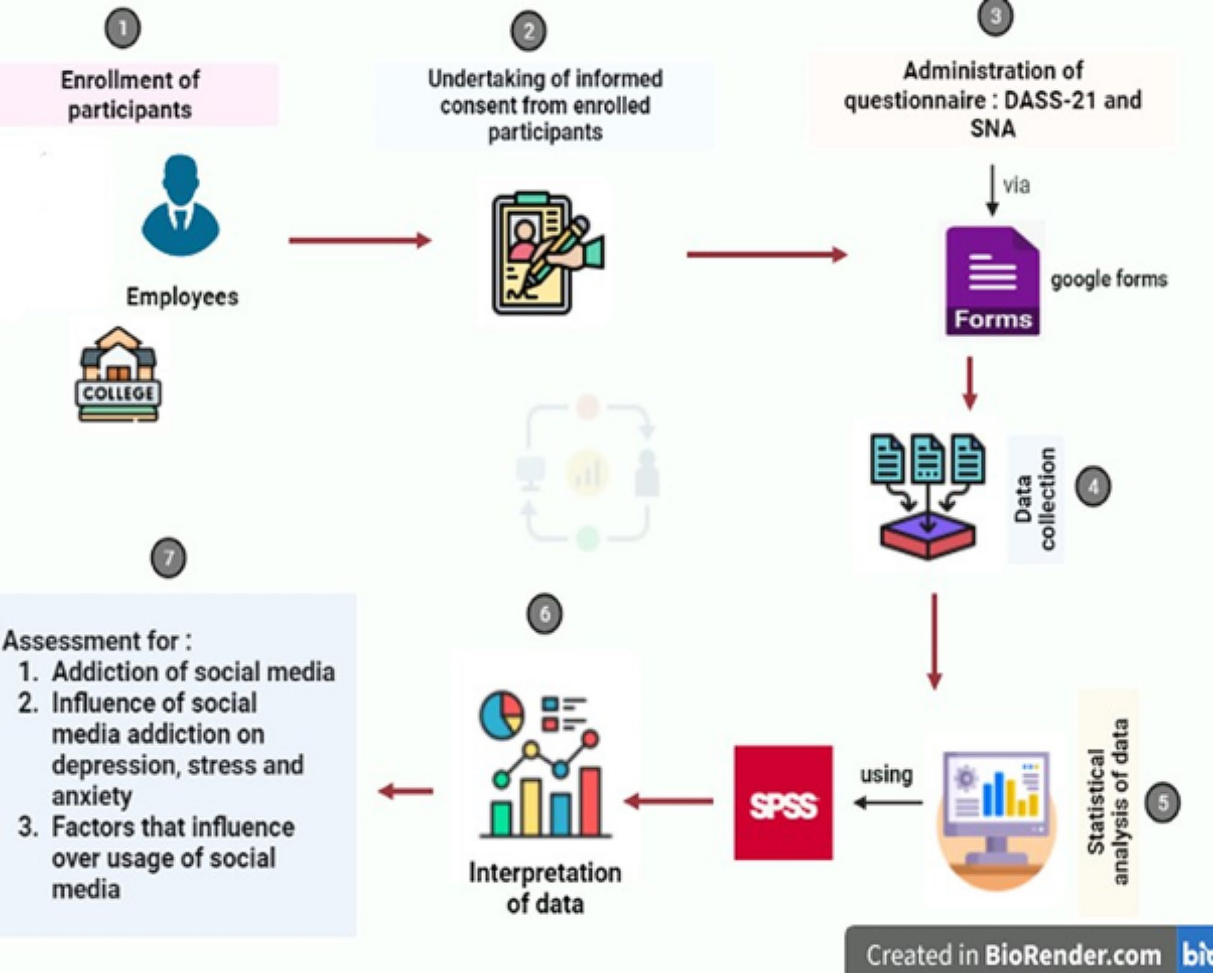
A pathway model analysis in the investigative study

An online survey had been conducted for the assessment of the impact of SMA on psychosocial behaviours such as depression, stress, and anxiety. A link to a structural questionnaire created with ‘Google Forms’, was sent to employees via WhatsApp and email. The response collection tool was built by a combination of two scales: the DASS-21 and social networking addiction scale (SNAS). Demographical information was also included in the questionnaire. Before participation, consent was obtained from employees, and all information provided was assured of confidentiality. The process of the study is depicted in Fig. 2. The questionnaire took an average of 5–10 min to complete. The form was completed by 205 employees (96 males and 109 females). When the forms were examined after the study, 5 forms were not evaluated due to incomplete data. Therefore, the data collection process was completed with 200 forms. The validity and reliability of questionnaire using the above-mentioned methods have been validated in the previous studies [17, 27, 28]. Therefore, the validity studies were not performed in the present study.

### Data collection tools

#### Sociodemographic characteristics

The survey sought questions about age, gender, and courses of employees (teaching and non-teaching), area of residence (Urban, suburban and rural), family type (joint and nuclear), marital status (married and unmarried), family income (lower, lower middle, upper class, upper lower, upper middle), social history (alcohol, tobacco), and mental health conditions and general health conditions, to create a profile of the participants’ sociodemographic characteristics.

#### DASS-21

The DASS-21, which has been used to assess mental health, was used to assess respondents’ mental health status. Three self-report measures are included in the DASS-21, which is intended to evaluate anxiety, stress, and depression. The seven elements on each of the three DASS-21 scales are broken down into subscales containing relevant data. According to Lovibond and Lovibond depression severity ratings range from 0 to 9, with 0 representing normal, 10–13 representing mild, 14–20 representing moderate, 21–27 representing severe, and 28 representing extremely severe. Anxiety has severity levels ranging from 0 to 7, with 0 being normal, 8–9 being mild, 10–14 being moderate, 15–19 being severe, and 20 or more being extremely severe. Stress is classified as 0–14 normal, 15–18 mild, 19–25 Moderate, 26–33 severe, and 34 or more extremely severe [29, 30].

#### SNAS

A 21-item Likert scale with seven possible responses was presented by Shahnawaz and Rehman in order to gauge how much a person experiences social network addiction. The possible range of the score is 21–147. A score of more than 84 denotes addiction, which is diagnosed at three different levels: mild, moderate, and severe. Factorial analysis of the scale is necessary to identify and evaluate the cultural and environmental factors that may influence these levels. People’s level of SMA can be determined and evaluated using the SNAS [31].

### Statistical analysis

The data was statistically analysed using the SPSS Statistics Base V 28 version of the Statistical Package for the Social Sciences software which is used to perform meta-analysis. Descriptive statistical methods (frequency, percentage, mean) were used to evaluate the study’s data which included the demographic details and SNAS and DASS-21 severity scoring; Chi-square analysis also called Pearson Chi-square analysis (a statistical test used to examine the differences between the categorical variables) which is a non-parametric test, was used to find the association of SMA with depression, stress, and anxiety. The results were assessed using a 95% confidence interval [32].

## Results

### Preliminary analysis

By using descriptive statistics like frequency and percentage of categorical data, a summary of the preliminary investigations on the demographic characteristics of the employees demonstrated potential covariates. The study population included majorly subjects of age above 25 years, as the most active demographics on SM. Responses received from the female subjects were more as compared to male respondents. Table 1 provides the summary of sociodemographic characteristics among the employee covariates and Table 2 provides a summary of the demographic details among employees in association with disorders.

**Table 1:** Sociodemographic characteristics among the employee

		Count	Column *N*%	Mean ± SEM
Age group	Below 35 years	103	50.2%	34.682 ± 0.60
	Above 35 years	102	49.8%	
Gender	Female	109	53.2%	102.5 ± 6.5
	Male	96	46.8%	
Designation	Non-teaching	108	52.7%	102.5 ± 5.5
	Teaching	97	47.3%	
Area of residence	Rural	11	5.4%	68.33 ± 42.77
	Sub-urban	42	20.5%	
	Urban	152	74.1%	
Family type	Joint	79	38.5%	102.5 ± 23.5
	Nuclear	126	61.5%	
Marital status	Married	138	67.3%	102.5 ± 35.5
	Single	67	32.7%	
Family income	Lower	1	0.5%	41 ± 29.63
	Lower middle	38	18.5%	
	Upper class	37	18.0%	
	Upper lower	2	1.0%	
	Upper middle	127	62.0%	
Social history	Alcohol	37	18.0%	68.33±49.86
	Nil	166	81.0%	
	Tobacco	2	1.0%	

Count = number of participants; N% = frequency by using descriptive statistics, SEM = standard error of mean

**Table 2:** Distribution of demographic details among employees in association with disorders

		Count	Column *N*%	Mean ± SEM
Rare disorders	Anaemia	1	0.5%	6.33 ± 3.17
	Migraine	6	6.3%	
	Obesity	12	5.9%	
Common disorders in employees	PCOD	4	2.0%	7 ± 3
	Thyroid	4	2.0%	
	Dysmenorrhea	13	2.9%	
Common disorders in employees	High blood pressure	58	28.3%	64 ± 20.42
	Diabetes	32	15.6%	
	None	102	49.8%	
Suffering from psychiatric disorder	Amnesia	8	3.9%	24.818 ± 9.31
	Anxiety disorder	43	21.0%	
	Mood disorder	74	36.1%	
	Attention deficit hyperactivity disorder	1	0.5%	
	Eating disorder	17	8.3%	
	Learning and Communication disorder	1	0.5%	
	None	87	42.4%	
	Suicidal thought disorder	2	1.0%	
	Job burnout	32	15.6%	
	Delirium	1	0.5%	
	Dementia	7	3.4%	

Count = Number of participants; N% = frequency by using descriptive statistics, SEM = standard error of mean

### SNAS and DASS-21 severity scoring

Descriptive statistics on SMA revealed that 30.2% of employees are addicted to SM. Cut-off scores for conventional severity labels (normal, moderate, severe) for depression, stress, and anxiety are statistically represented using frequency and percentage (Table 3).

**Table 3:** Distribution of SNAS, depression, anxiety, and stress among employees

		Count	Column *N*%	Mean ± SEM
SNAS	No Addiction	46	22.4%	102.5 ± 56.5
	Addicted	159	77.6%	
Depression	Normal	93	45.4%	41 ± 14.43
	Mild	20	9.8%	
	Moderate	23	11.2%	
	Severe	17	8.3%	
	Extremely Severe	52	25.4%	
Anxiety	Normal	61	29.8%	41 ± 12.84
	Mild	18	8.8%	
	Moderate	36	17.6%	
	Severe	11	5.4%	
	Extremely Severe	79	38.5%	
Stress	Normal	98	47.8%	41 ± 14.69
	Mild	18	8.8%	
	Moderate	20	9.8%	
	Severe	32	15.6%	
	Extremely Severe	37	18.0%	

Count = Number of participants; N% = frequency by using descriptive statistics, SEM = standard error of mean

### Main analysis

#### Association of depression, anxiety and stress with SMA

A χ² test was run to compare depression, anxiety and stress with SNAS. Since all predicted cell frequencies were higher than 5, the χ² test’s assumptions were satisfied.

#### Association of depression with SMA

Between SMA and depression, there was no statistically significant correlation (χ² (4) = 4.09, *p* = 0.394). The calculated *p*-value of 0.394 is above z the defined significance level of 5%. As seen in Table 4, the null hypothesis is not rejected and the χ² test is not significant.

**Table 4:** Association of depression with SMA among addicted and non-addicted employee

		SMA				χ2 value	*P* value	Mean ± SEM (No addiction)	Mean ± SEM (Addiction)
		No addiction		Addiction					
		*N*	%	*N*	%				
Depression	Normal	33	71.70%	60	37.70%	24.598	< 0.001*	9.2 ± 6.036	31.8 ± 9.78
	Mild	5	10.90%	15	9.40%				
	Moderate	6	13.00%	17	10.70%				
	Severe	1	2.20%	16	10.10%				
	Extremely severe	1	2.20%	51	32.10%				

*Statistical significance was set at 0.05; N: No. of samples; χ2: Chi-square; SMA: social media addiction; SEM: standard error of mean

#### Association of anxiety with SMA

Between SMA and anxiety, there was no statistically significant correlation (χ² (4) = 2.02, *p* = 0.732). The calculated *p*-value of 0.732 is above the defined significance level of 5%. As seen in Table 5, the null hypothesis is not rejected and the χ² test is not significant.

**Table 5:** Association of anxiety with SMA among addicted and non-addicted employee

		SMA				χ2 value	*P* value	Mean ± SEM (No addiction)	Mean ± SEM (Addiction)
		No addiction		Addicted					
		*N*	%	*N*	%				
Anxiety	Normal	23	50.00%	38	23.90%	20.33	< 0.001*	9.2 ± 3.652	31.8 ± 11.54
	Mild	3	6.50%	15	9.40%				
	Moderate	10	21.70%	26	16.40%				
	Severe	4	8.70%	7	4.40%				
	Extremely severe	6	13.00%	73	45.90%				

*Statistical significance was set at 0.05; N: No. of samples; χ2: Chi-square; SMA: social media addiction; SEM: standard error of mean

#### Association of stress with SMA

Between SMA and stress, there was no statistically significant correlation (χ² (4) = 0.39, *p* = 0.983). The calculated *p*-value of 0.983 is above the defined significance level of 5%. As seen in Table 6, the null hypothesis is not rejected and the χ² test is not significant.

**Table 6:** Association of stress with SMA among addicted and non-addicted employee

		SMA				χ2 value	*P* value	Mean ± SEM (No addiction)	Mean ± SEM (Addiction)
		No addiction		Addicted					
		*N*	%	*N*	%				
Stress	Normal	33	71.70%	65	40.90%	18.889	< 0.001*	9.2±5.98	31.8±9.30
	Mild	5	10.90%	13	8.20%				
	Moderate	4	8.70%	16	10.10%				
	Severe	3	6.50%	29	18.20%				
	Extremely severe	1	2.20%	36	22.60%				

*Statistical significance was set at 0.05; N: No. of samples; χ2: Chi-square; SMA: social media addiction; SEM: standard error of mean

### Conceptual structure of factors influencing the overuse of SM in employees

It was found that employees excessively overused SM for enjoyment, to build relationship, to prevent boredom, loneliness and avoid exhaustion, for satisfaction, peer/parent influence and for continual knowledge and information exchange. This has been denoted in Table 7.

**Table 7:** Factors influencing the overuse of social media in employees

		Count	Column *N* %	Mean ± SEM
Factors influencing the overuse of social media	Constant knowledge and information sharing	67	32.7%	56.1 ± 10.69
	For entertainment	126	61.5%	
	Relationship	77	37.6%	
	To avoid exhaustion	40	19.5%	
	To fill up spare time	55	26.8%	
	For recognition	0	0.0%	
	To avoid boredom	79	38.5%	
	For satisfaction	44	21.5%	
	Peer/parent influence	36	17.6%	
	To overcome loneliness	37	18.0%	

Count = Number of participants; N% = frequency by using descriptive statistics

## Discussion

Recent years have witnessed a significant expansion in the use of SM, impacting daily life globally [1, 3]. Recent studies highlight a sharp rise in SM usage, sparking interest in this subject and potential drawback [3]. Increased use, particularly on social networking sites, raises the risk of addiction, termed “problematic social media use” [1–3]. This overuse hinders regular functioning, characterized by addictive traits [4]. This is true despite the many benefits of technology, such as easy access to information, effortless interaction with communities around the world, entertainment, and business development. When utilising SM platforms, people may experience a wide range of emotions both positive and negative, which may have an effect on their mental health [1–5].

In primary care settings, depression, stress, and anxiety are all common illnesses with a high risk of co-occurrence [3, 10, 13]. Depression and anxiety have been connected to negative emotional SM experiences [10]. There is significant discussion over the impact of pleasant feelings and distraction on users’ mental health [12]. While some researchers caution against the potential harms of online use, others advise against overstating its effects on mental health [13–15]. Therefore, this study’s outcomes may help in some manner to clarify the important controversy over inappropriate SM consumption. A few of the significant issues that depression is linked to are incomplete education, a higher incidence of unwanted pregnancies, significantly fewer affluent interpersonal connections, and an increased risk of drug abuse and suicidality. Depressed people are also more susceptible to being ingested by SM [15–17]. Stress is a defence mechanism that humans have developed as a means of enabling us to act quickly in the face of acute threats. On the other side, persistent stress can result in physical and mental chronic disorders [29]. Stress is defined as a sensation accompanied with predictable biochemical, physiological, and behavioural changes [29, 30]. There are several hypotheses explaining how using SM may lead to stress, and these changes in the body’s psychological makeup might be brought on by doing so [29–32]. This study examined the relationship between SMA and psychosocial behaviours including depression, stress, and anxiety in employees, and identified the key characteristics that led the group to use SM excessively. SM usage and mental health issues are often linked, even if our study’s results were not totally consistent.

Due to the fact that employees tend to overuse cell phones, we gathered the data using the DASS-21 and SNAS. Second, this study examine how SM affected employees’ psychosocial behaviours. Due to the fact that any individual’s demographic traits have a significant impact on the study’s findings, we have gathered a variety of demographic information from employees. First, more female employees gave replies than male employees did (Table 1), and most of the respondents were from metropolitan areas. A nuclear family made up 70% of them. Additionally, this study included a list of general health issues, which also includes mental health issues. Psychological issues may be attributed to people with any number of mental diseases, which may turn out to be the primary cause of SMA.

The SNAS was used to gauge the extent of addiction among the employees, and the findings showed that roughly 77.6% of the staff members had severe SM addiction (Table 3). This must have been caused due to onset of social anxiety. Health line recommends certain therapies for a such population to overcome their addiction. The therapies include; cognitive behavioural therapy, motivational interviewing, and group counselling sessions. The DASS-21 answers revealed that the majority of employees fell into the normal range, indicating that the rest of the population had depression scores ranging from mild to severely severe (Table 3). Numerous factors, including FOMO, academic pressure, poor sleep, financial difficulties, drug abuse, and loneliness, among others, may contribute to this. The findings of the Chi-square test used to analyse the link between depression, stress, anxiety, and SMA showed that the level of depression, stress, and anxiety, as well as the range of addiction to SM observed in employees, has been high and has demonstrated statistical significance (*p* = 0.001). These observations were in good agreement with those pointed out in the previous studies [2, 6, 9, 11, 27, 28].

Additionally, it was found that employees heavily overused SM for enjoyment, to prevent boredom, and for continual knowledge and information exchange. To provide a more focused assessment, further future research is needed to look into the etiological pathways connecting SMA with psychological issues. Additionally, more investigation is required to determine the effect of SM on sleep quality and semantic memory.

## Conclusion

In conclusion, this study demonstrated a significant association between SMA and psychosocial factors like depression, stress, and anxiety among the employee population. The health line elucidates certain therapies to treat these conditions; such as Psychotherapies, Alternative therapies like herbal remedies, massage, acupuncture, yoga, and medication. Additionally, to overcome addiction to SM, employees are recommended to seek help by obtaining preventive therapies. Responses collected from the population has shed information that the prime factors that led them to overuse SM were entertainment and to avoid boredom.

## Summary

SM facilitates the development of relationships among individuals with similar backgrounds and interests. SMA is characterized by excessive worry, an insatiable need for access, and a commitment that interferes with other life aspects. While most users engage harmlessly, a small percentage exhibits addictive behaviour, leading to negative impacts on productivity and mental health, including depression, anxiety, and stress. The impact of excessive SM use is explored, including its link to the productivity issues among workers, and its negative effects on mental health, such as depression, anxiety, and stress. This study demonstrated a significant association between SMA and psychosocial factors like depression, stress, and anxiety among the employee population.

### Electronic supplementary material

Below is the link to the electronic supplementary material.

Supplementary Material 1

## Data Availability

No datasets were generated or analysed during the current study.

## Abbreviations

SM: Social media
SMA: Social media addiction
SNAS: Social networking addiction scale
DASS-21: Depression, anxiety, and stress scale-21
SPSS: Statistical product and service solutions
FOMO: Fear of missing out
JSS AHER: JSS Academy of Higher Education and Research
