# Supplementary material for: Assessment of the impact of social media addiction on psychosocial behaviour like depression, stress, and anxiety in working professionals
Source: BMC Psychol. 2024 Jun 15;12:352. doi: 10.1186/s40359-024-01850-2 (PMC11179207; doi:10.1186/s40359-024-01850-2)
Supplement: Supplementary file 1 — Supplementary Material 1 [file 40359_2024_1850_MOESM1_ESM.pdf]

https://drive.google.com/dr...

EMPLOYEE FORM

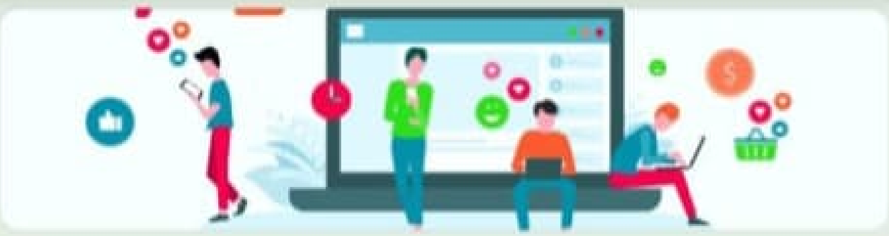

# An Assessment of the Impact of Social Media Addiction on Psychosocial Behaviour like Depression, Anxiety, and Stress.

* Indicates required question

[illegible] *

Willing to participate in this survey? *

- ○ Yes
- ○ No

2. Age *

3. Gender *

- ○ Male
- ○ Female

https://drive.google.com/dr...

EMPLOYEE FORM

4. Designation *

- Teaching
- Non-teaching

5. Area of residence *

- Urban
- Sub-urban
- Rural

6. Family type *

- Joint
- Nuclear

https://drive.google.com/dr...

EMPLOYEE FORM

7. Marital status *

- ○ Married
- ○ Single

8. Family income *

- ○ Upper class
- ○ Upper middle
- ○ Lower middle
- ○ Upper lower
- ○ Lower

9. Social history *

9. Social history *

- ◯ Alcohol
- ◯ Tobacco
- ◯ Nil

10. Suffering from any of the below disorders? *

- ☐ Diabetes
- ☐ High BP
- ☐ Low BP
- ☐ Thyroid
- ☐ PCOD
- ☐ Migraine
- ☐ Obesity

11. Suffering from any of the below mental disorders? *

- [ ] Eating disorder
- [ ] Suicidal thought disorder
- [ ] Amnesia
- [ ] Mood disorder
- [ ] Anxiety disorder
- [ ] Learning and communication disorder
- [ ] Dementia
- [ ] Delirium
- [ ] Job burnout
- [ ] None

1. While I work/ study, my mind remains on social networking sites. *

- ◯ Strongly Disagree
- ◯ Disagree
- ◯ Somewhat Disagree
- ◯ Neither Agree nor Disagree
- ◯ Somewhat Agree
- ◯ Agree
- ◯ Strongly Agree

2. I go to social networking sites instantly after waking up in the morning. *

https://drive.google.com/dr...

My Drive

○ Strongly Agree

2. I go to social networking sites instantly after waking up in the morning. *

○ Strongly Disagree

○ Disagree

○ Somewhat Disagree

○ Neither Agree nor Disagree

○ Somewhat Agree

○ Agree

○ Strongly Agree

3. I check for updates on social networking sites while *

Submitted by:
[illegible]

https://drive.google.com/dr...

My Drive

3. I check for updates on social networking sites while studying/working. *

- ○ Strongly Disagree
- ○ Disagree
- ○ Somewhat Disagree
- ○ Neither Agree nor Disagree
- ○ Somewhat Agree
- ○ Agree
- ○ Strongly Agree

4. I check my social networking account before starting any task or activity. *

Submitted by:
Vaishnavi Jahagirdar
M.Pharm 1st Year

https://drive.google.com/dr...

EMPLOYEE FORM

5. I go to social networking sites whenever I am upset. *

- Strongly Disagree
- Disagree
- Somewhat Disagree
- Neither Agree nor Disagree
- Somewhat Agree
- Agree
- Strongly Agree

6. Social networking helps me lift my mood. *

- Strongly disagree
- Disagree

- Somewhat disagree
- Neither agree or disagree
- Somewhat agree
- Agree
- Strongly agree

7. I feel relaxed whenever I am on social networking sites *

- Strongly disagree
- Disagree
- Somewhat disagree
- Neither agree nor disagree
- Somewhat agree
- Agree
- Strongly agree

https://drive.google.com/dr...

My Drive

○ Strongly agree

8. These days I spend more and more time on social networking sites *

○ Strongly disagree
○ Disagree
○ Somewhat disagree
○ Neither agree nor disagree
○ Somewhat agree
○ Agree
○ Strongly agree

9. When compared I spend *

Strongly agree

9. When compared I spend more time on social networking sites now than I did in the past. *

- Strongly disagree
- Disagree
- Somewhat disagree
- Neither agree nor disagree
- Somewhat agree
- Agree
- Strongly agree

https://drive.google.com/dr...

My Drive

[illegible]

○ Strongly agree

10. I need to be on social networking sites for longer time than before to be satisfied *

○ Strongly disagree

○ Disagree

○ Somewhat disagree

○ Neither agree nor disagree

○ Somewhat Agree

○ Agree

○ Strongly agree

Submitted by:

- Agree
- Strongly agree

11. I feel sad when I am unable to log in to social networking sites. *

- Strongly disagree
- Disagree
- Somewhat disagree
- Neither agree nor disagree
- Somewhat agree
- Agree
- Strongly agree

https://drive.google.com/dr...

My Drive

Agree

Strongly agree

12. I become irritable whenever I cannot log in to social networking sites *

Strongly disagree

Disagree

Somewhat disagree

Neither agree nor disagree

Somewhat agree

Agree

Strongly agree

https://drive.google.com/dr...

My Drive

13. I feel frustrated when I cannot use social networking sites. *

- Strongly disagree
- Disagree
- Somewhat disagree
- Neither agree nor disagree
- Somewhat agree
- Agree
- Strongly agree

14. I become restless when I do not get time for social *

https://drive.google.com/dr...

My Drive

Agree

Strongly agree

14. I become restless when I do not get time for social networking. *

Strongly disagree

Disagree

Somewhat disagree

Neither agree nor disagree

Somewhat agree

Agree

Strongly agree

https://drive.google.com/dr...

My Drive

○ Somewhat agree

○ Agree

○ Strongly agree

15. I try to hide the time I spend on social networking *

○ Strongly disagree

○ Disagree

○ Somewhat disagree

○ Neither agree nor disagree

○ Somewhat agree

○ Agree

○ Strongly agree

https://drive.google.com/dr...

My Drive

16. I need to lie to my parents and others when they ask about my social networking usage. *

- Strongly disagree
- Disagree
- Somewhat disagree
- Neither agree nor disagree
- Somewhat agree
- Agree
- Strongly agree

17. I ignore my sleep because I have/want to be on social *

https://drive.google.com/dr...

My Drive

- Agree
- Strongly agree

17. I ignore my sleep because I have/want to be on social networking sites. *

- Strongly disagree
- Disagree
- Somewhat disagree
- Neither agree nor disagree
- Somewhat Agree
- Agree
- Strongly agree

- Agree
- Strongly agree

18. I have failed to cut down the time I spend on social networking sites. *

- Strongly disagree
- Disagree
- Somewhat disgaree
- Neither agree nor disgaree
- Somewhat agree
- Agree
- Strongly agree

Somewhat agree

Agree

Strongly agree

19. I have tried to stop using social networking sites, but have failed. *

Strongly disagree

Disagree

Somewhat disagree

Neither agree nor disagree

Somewhat Agree

Agree

Strongly agree

https://drive.google.com/dr...

My Drive

- ○ Agree
- ○ Strongly agree

20. I am unable to cut-down the time I spend on social networking sites. *

- ○ Strongly disagree
- ○ Disagree
- ○ Somewhat disagree
- ○ Neither agree nor disagree
- ○ Somewhat agree
- ○ Agree
- ○ Strongly agree

Submitted by:
Vaishnavi Jahagirdar

https://drive.google.com/dr...

My Drive

Strongly agree

21. **My repeated attempts to reduce the time I spend on social networking sites have failed** *

- Strongly disagree
- Disagree
- Somewhat disagree
- Neither agree nor disagree
- Somewhat agree
- Agree
- Strongly agree

https://drive.google.com/dr...

My Drive

Select the factors or reasons that have led you to overuse social media *

- [ ] To overcome Loneliness
- [ ] For Satisfaction
- [ ] Peer influence
- [ ] To avoid Boredom
- [ ] For Entertainment
- [ ] Relationship
- [ ] To avoid exhaustion
- [ ] To fill up spare time
- [ ] Constant knowledge and information sharing

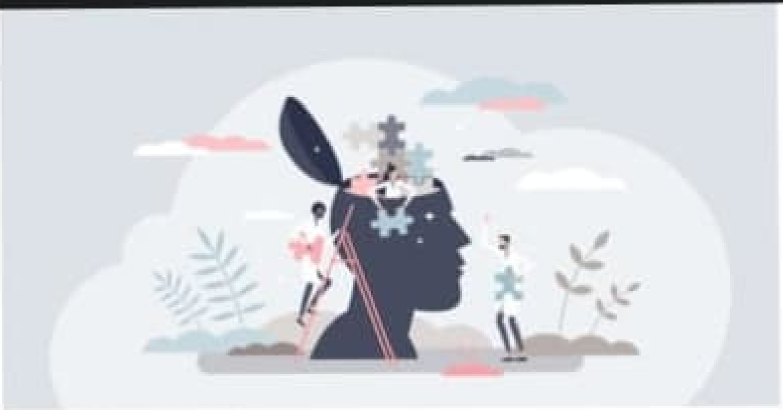

Please read each statement and tick a number 0,1,2 or 3 which indicates how much the statement is applied to you

The rating scale is as follows:

0 Did not apply to me at all

1 Applied to me to some degree, or some of the time

2 Applied to me to a considerable degree or a good part of time

3 Applied to me very much or most of the time

https://drive.google.com/dr...

My Drive

some of the time
2 Applied to me to a considerable degree or a good part of time
3 Applied to me very much or most of the time

1. I found it hard to wind down (difficult to relax after stress) *

0 ○
1 ○
2 ○
3 ○

2. I was aware of dryness of *

https://drive.google.com/dr...

My Drive

2. I was aware of dryness of my mouth *

0 ○

1 ○

2 ○

3 ○

3. I couldn't seem to experience any positive feelings at all *

0 ○

1 ○

2 ○

3 ○

https://drive.google.com/dr...

My Drive

4. I experienced breathing difficulty *

0 ○

1 ○

2 ○

3 ○

5. I found it difficult to work up the initiative to do things *

0 ○

1 ○

2 ○

3 ○

Submitted by:
Vaishnavi Jahagirdar

6. I tended to overreact to situations *

0
1
2
3

7. I experienced trembling (shiver - e.g. in the hands) *

0
1
2
3

https://drive.google.com/dr...

My Drive

8. I felt that I was using a lot of nervous energy *

0 ○

1 ○

2 ○

3 ○

9. I was worried about situations in which I might panic and make a fool of myself *

0 ○

1 ○

2 ○

10. I felt that I had nothing to look forward to *

0 ○

1 ○

2 ○

3 ○

11. I found myself getting agitated (feeling troubled) *

0 ○

1 ○

2 ○

3 ○

12. I found it difficult to relax *

- 0 ○
- 1 ○
- 2 ○
- 3 ○

13. I felt down-hearted and blue *

- 0 ○
- 1 ○
- 2 ○

https://drive.google.com/dr...

My Drive

14. I was intolerant of anything that kept me from getting on with what I was doing *

0 ○

1 ○

2 ○

3 ○

15. I felt I was close to panic *

0 ○

1 ○

2 ○

3 ○

16. I was unable to become enthusiastic about anything *

0 ○

1 ○

2 ○

3 ○

17. I felt I wasn't worth much as a person *

0 ○

1 ○

2 ○

3 ○

18. I felt that I was rather touchy (cranky, quick tempered) *

0 ○

1 ○

2 ○

3 ○

19. I was aware of the action of my heart in the absence of physical exertion (e.g. sense of heart rate increase) *

0 ○

1 ○

2 ○

https://drive.google.com/dr...

My Drive

20. I felt scared without any good reason *

0 ○

1 ○

2 ○

3 ○

21. I felt that life was meaningless *

0 ○

1 ○

2 ○

3 ○

Submitted by:
